# Chronicity of self-harming behaviors among adolescent teenage girls living in refugee settlements in Northern Uganda

**DOI:** 10.1186/s40359-024-01897-1

**Published:** 2024-07-16

**Authors:** Mark Mohan Kaggwa, Joan Abaatyo, Donald Otika, Pebalo Francis Pebolo, Felix Bongomin

**Affiliations:** 1 Forensic Psychiatry Program, St Joseph’s Healthcare, Hamilton, ON Canada; 2 Department of Psychiatry and Behavioral Neurosciences, McMaster University, Hamilton, Canada; 3 Department of Psychiatry, Faculty of Medicine, Uganda Christian University, Kampala, Uganda; 4 King Ceasor University, Kampala, Uganda; 5 Faculty of Medicine, Gulu University, Gulu, Uganda; 6 Department of Reproductive Health, Faculty of Medicine, Gulu University, Gulu, Uganda; 7 Department of Medical Microbiology and Immunology, Faculty of Medicine, Gulu University, Gulu, Uganda; 8 Department of Internal Medicine, Gulu Regional Referral Hospital, Gulu, Uganda

**Keywords:** Self-harm ideations, Chronicity of self-harming behaviors, suicidal behaviors, teenagers, Females, Refugees, and Uganda

## Abstract

**Background:**

Self-harming ideations demand targeted research due to their persistent nature, especially among female adolescents within refugee populations who face unique challenges that can exacerbate self-harming tendencies. This study aimed to assess the factors associated with self-harming ideations chronicity among female teenagers living in refugee settlement in Northern Uganda.

**Method:**

This cross-sectional study used a pretested questionnaire to assess self-harming ideations and other demographic characteristics. Ordinal logistic regression was used to determine factors associated with chronicity of self-harm ideations.

**Results:**

Of 385 participants, the prevalence of self-harming ideations was 4.2% (*n* = 16) for acute, 8% (*n* = 31) for subacute, and 3.1% (*n* = 12) for chronic. The likelihood of having more chronic self-harming ideations increased with having ever been pregnant (adjusted odds ratio [aOR] = 3.78, 95% Confidence Interval [CI] = 1.57–9.08). However, having a spouse as the family head reduced the likelihood of having more chronic self-harming ideations (aOR = 0.19, 95% CI = 0.04–0.95).

**Conclusions:**

The persistence of self-harming thoughts among female teenagers in Northern Ugandan refugee settlements varies. Pregnancy history is associated with a higher chance of prolonged self-harming thoughts while having a spouse as the family’s head is linked with a lower likelihood. Examining different demographic and familial elements when addressing the mental well-being of female teenage refugees is vital. It stresses the necessity for customized interventions and support networks targeting the reduction of self-harm behaviors among this vulnerable group.

**Supplementary Information:**

The online version contains supplementary material available at 10.1186/s40359-024-01897-1.

## Introduction

Self-harming behaviors, a manifestation of profound emotional distress, have garnered increased attention in recent years due to their complex and often chronic nature [1]. The persistence of self-harming thoughts warrants attention due to its significant impact on the mental well-being of individuals grappling with such tendencies [2]. These persistent thoughts intensify psychological distress, giving rise to anxiety, depression, a pervasive sense of hopelessness, and suicidality [3, 4]. The resulting heightened isolation, stemming from associated stigma and strained relationships, further hampers daily functioning, leading to concentration difficulties, disrupted sleep patterns, and maladaptive coping mechanisms [3]. Despite extensive research shedding light on self-harm prevalence and risk factors, a comprehensive understanding of the persistence of these behaviors remains elusive, particularly among vulnerable populations like teenage refugee girls, who are susceptible to the enduring effects of conflict and displacement.

Adolescence, being a pivotal developmental stage, coupled with the unique challenges faced by the refugee population, may contribute to the perpetuation of self-harming ideations, especially among females [5–9]. The enduring global concern for refugees, particularly in areas characterized by conflict and displacement, like Northern Uganda, is undeniable [10, 11]. Northern Uganda, characterized by a tumultuous history of conflict and displacement, hosts a significant refugee population [12, 13]. Despite this, the region contends with limitations in mental health services specifically designed for refugees [14]. Research shows that refugees face a higher risk of self-harm due to a mix of pre-migration trauma, like violence and persecution, and post-migration challenges, such as asylum uncertainty, cultural adjustment issues, and economic instability [15, 16]. A study in Italy showed the prevalence of self-harm in this demographic to be 26.6% [17]. Common mental health problems among refugees, including depression, anxiety, and PTSD, increase the likelihood of self-harm. Additionally, difficulties in accessing mental health care, including language barriers, stigma, and the absence of culturally sensitive services, further heighten their vulnerability [17–19]. In addition, the refugee populations are encompassed in severe economic challenges, like limited job opportunities and financial instability that increase their chances of despair and having chronic thoughts of preferring to die [20, 21]. The refugees are also in a new environment with many individuals from different cultures, breaking the protective cultural bond and sense of belonging. This cultural misalignment might lead to feelings of isolation, exacerbating mental health difficulties and potentially increasing the risk of persistent self-harm ideations [21]. The cultural and language differences might lead to difficulties in providing psychoeducation and proper mental healthcare, thus perpetuating persistent self-harming behaviors [20, 21]. Consequently, the enduring consequences of prolonged conflicts cast an indelible mark on the mental well-being of the area’s residents [14].

The persistence of self-harming ideations among female teenage refugees may be influenced by a complex interplay of factors. Increasing age and higher education levels are generally associated with the development of healthier coping mechanisms, better emotional regulation, and improved access to mental health information, which can contribute to a reduction in the chronicity of self-harm ideations [22–24]. However, most females living in refugee settlements are deprived of pursuing further studies and are already lagging in their academic journey due to the escaped insurgencies. On the other hand, psychological distress, which can be exacerbated by poor social support structures and economic disadvantages, such as those within refugee camps, have been linked to an increased persistence of self-harm ideations [5, 6, 25]. Additionally, adolescent sexual and reproductive health challenges, including lack of education and information on sexual risks, early pregnancy, restricted access to services, and stigma surrounding sexual health, may also heighten the risk of persistent self-harm ideations [26–31]. Not underscoring the other causes of persistent self-harming behaviors, we acknowledge that the causes are multifactorial and are an interplay of various factors that need comprehensive support systems to address them among this vulnerable population.

Understanding the factors contributing to the persistence of self-harming behaviors is crucial for developing targeted interventions and support systems. This study, therefore, sought to assess the factors associated with self-harming ideations chronicity among females living in refugee settlements in Northern Uganda.

## Methods

Between March and June 2023, we conducted a community-based, cross-sectional study in four randomly selected refugee settlements of participants who migrated to Northern Uganda. Based on the Kish and Lisle (1965) formula for calculation of sample size for an unknown population, the study enrolled a total of 385 adolescent girls living in these settlements. This region hosts a refugee population of 626,331, of whom women and children constitute 82%. However, there is no data specific to the population of teenage girls [32]. Ethical approval was obtained from the Gulu University Research and Ethics Committee (GUREC-2022-291) and appropriate relevant permissions were obtained.

The study employed a multi-stage sampling approach to randomly select two out of the five districts in Northern Uganda with refugee settlements. The districts—Adjumani, Arua, Koboko, Obongi, and Yumbe—were listed on pieces of paper, mixed, and two were randomly chosen. Additionally, cluster sampling was used to identify two refugee settlements from each chosen district. Each settlement represented a cluster, resulting in a total of four clusters. This random selection aimed to prevent bias and ensure reliable results.

For participant selection, we conveniently included only female respondents aged 15 to 19. Consent to participate was obtained from those 18 -19years, from the parents or legal guardians for participants below age of 18 and following approval, assent was obtained. Participants who reported prior diagnosis of severe major mental illnesses including schizophrenia, bipolar disorder and major depressive disorder, were excluded from the study.

Trained research assistants were recruited and underwent a one-day training on the research tool, research ethics, and good clinical practice. They collected data, explained the study’s purpose to identify respondents, and obtained informed consent. The questionnaire was administered through an electronic form stored in the Kobo toolbox mobile application.

### Study measures

We developed a semi-structured questionnaire pretested among respondents of similar characteristics outside the study area. The questionnaire had different sections, including the sociodemographic details (age, marital status, level of education, employment status, monthly income, media exposure, sex of family head, relationship with family head), prior pregnancy history, intent to get pregnant in 6 months, current contraceptive use and self-harm ideations.

Self-harm ideation chronicity was assessed as follows. First, participants were screened for recent/acute self-harming behaviors: “*During the past two weeks*,* have you had thoughts of hurting yourself*?” Those who screened positive were also asked for a history of experiencing self-harming behaviors beyond the recent two weeks but less than one month (subacute): “*Has there been a time in the past month where you had thoughts of ending your life*?” Those who screened positive were asked for experience of self-harming behaviors beyond one month, i.e., chronic self-harming behaviors. The scale reliability coefficient of this tool was 0.60.

### Data analysis

Data was analyzed using STATA version 15. Categorical variables were summarized using frequencies and percentages, while the nonparametric continuous variable, age, was summarized using median and interquartile range (IQR). Bivariate and multivariate ordinal logistic regression analyses were performed to assess associations with chronicity of suicidal ideations. Factors with a p-value of < 0.2 at bivariate analysis were tested for collinearity using variance inflation factors (VIF). Those with a VIF below three were included in the final model at multiple logistic regression. The significant level was at less than 5% for a 95% confidence interval.

## Results

### Participant characteristics

The study involved 385 teenage girls residing in refugee settlements. Their median age was 17, with an interquartile range (IQR) of 15 to 18 years. The majority (82.1%, 316) had completed primary education at their highest level, and 85.6% (*n* = 329) were unemployed. Approximately 22.1% (*n* = 85) identified their spouses as household leaders, and 34% (*n* = 131) had experienced pregnancy.

### Self-harming behaviors

The distribution of participants across self-harm ideations chronicity was as follows: acute (4.2%), subacute (8.0%), and chronic (3.1%).

### Factors associated with chronicity of self-harm ideations among girls living in refugee settlements of Northern Uganda

At the bivariate analysis level, an increase in age, having primary education as the highest level of education, having media exposure, having ever been pregnant, and having a male as the head of the family were statistically significantly associated with an increased likelihood of having more chronic self-harming ideations. However, having a parent as the family head reduced the likelihood of having more chronic self-harming ideations.

In the multivariate analysis, the likelihood of having more chronic self-harming ideations increased with having ever been pregnant (adjusted odds ratio (aOR) = 3.78, 95% confidence interval CI) = 1.57–9.08). Conversely, having a spouse as the family head (aOR = 0.19, 95% CI = 0.04–0.95) reduced the likelihood of having more chronic self-harming ideations (see Table 1).

**Table 1:** Participant characteristics distribution across chronicity of experiencing self-harm ideations

Variable	Self-harm				Bivariate analysis	Multivariable analysis
	None 326 (84.7%)	Acute (within the past two weeks only) 16 (4.2%)	Subacute (within the past one month) 31 (8.0%)	Chronic (more than one month) 12 (3.1%)	Crude Odds ratio [cOR] (95% Confidence Interval [CI))	Adjusted Odds ratio [aOR (95% CI)]
Age (Median, IQR)	17 (15–18)	16 (15–18)	18 (18–19)	18 (17.5–18)	1.46 (1.19–1.78) **	1.25(0.93–1.67)
**Level of education**						
None	3 (0.9)	0	1 (3.2)	1 (8.3)	1 (reference)	1 (reference)
Primary	273 (83.7)	14 (87.5)	21 (67.7)	8 (66.7)	0.18(0.03 − 1.14) *	0.41 (0.06–2.71)
Secondary and tertiary	50 (15.3)	2 (12.5)	9 (29.0)	3 (25.0)	0.34 (0.05–2.24)	0.46 (0.06–3.32)
**Marital status**						
Married	77 (23.6)	1 (6.3)	10 (32.3)	8 (66.7)	1 (reference)	1 (reference)
Unmarried	249 (76.4)	15 (93.7)	21 (67.7)	4 (33.3)	0.59 (0.32–1.07)	1.27 (0.29–5.47)
**Employment status**						
Employed	281 (86.2)	11 (68.7)	25 (80.7)	12 (100)	1 (reference)	
Unemployed	45 (13.8)	5 (31.3)	6 (19.3)	0	1.32 (0.64–2.70)	
**Have media exposure** *(listens to radio OR owns mobile phone OR watches television OR reads Newspaper)*						
No	165 (50.6)	5 (31.3)	8 (25.8)	6 (50.0)	1 (reference)	1 (reference)
Yes	161 (49.4)	11 (68.8)	23 (74.2)	6 (50.0)	2.10 (1.17–3.78) *	0.41 (0.74–2.70)
**Monthly income**						
</50,000UGX	317 (97.2)	15 (93.8)	30 (96.8)	12 (100)	1 (reference)	
>50,000UGX	9 (2.8)	1 (6.2)	1 (3.2)	0	1.15 (0.25–5.36)	
**Ever been pregnant**						
No	230 (70.6)	11 (68.8)	11 (35.5)	2 (16.7)	1 (reference)	1 (reference)
Yes	96 (29.4)	5 (31.2)	20 (64.5)	10 (83.3)	3.76 (2.12–6.64) **	3.78 (1.57–9.08) *
**Intent to get pregnant in 6months**						
No	306 (93.9)	16 (100)	30 (96.8)	11 (91.7)	1 (reference)	
Yes	20 (6.1)	0	1 (3.2)	1 (8.3)	0.56 (0.13–2.48)	
**Current contraceptive use**						
No	25 (7.7)	0	3 (9.7)	1 (8.3)	1 (reference)	
Yes	301 (92.3)	16 (100)	28 (90.3)	11 (91.7)	0.87 (0.40–1.89)	
**Sex of family head**						
Female	186 (57.1)	7 (43.8)	14 (45.2)	4 (33.3)	1 (reference)	1 (reference)
Male	140 (42.9)	9 (56.2)	17 (54.8)	8 (66.7)	1.82 (1.04–3.18) *	1.90 (0.97–3.75)
**Relationship with family head**						
Other relative	37 (11.3)	5 (31.3)	5 (16.1)	2 (16.7)	1 (reference)	1 (reference)
Spouse	69 (21.2)	1 (6.3)	9 (29.0)	6 (50.0)	0.81 (0.35–1.87)	0.19 (0.04–0.95) *
Parent	220 (67.5)	10 (62.5)	17 (54.8)	4 (33.3)	0.45 (0.21–0.95) *	0.45 (0.20–1.01)

## Discussion

This study examined the chronicity of self-harming behaviors among female teenagers living in refugee settlements in Northern Uganda. The results indicate that 4.2% of female teenage refugees have acute self-harm ideations, 8% have subacute, and 3.1% have chronic self-harm ideations. The likelihood of having more chronic self-harming ideations increased with having ever been pregnant. However, having a spouse as the family head reduced the likelihood of having more chronic self-harming ideations.

The prevalence of self-harm ideations in this study, observed at acute, sub-acute, and chronic stages, was found to be less frequent compared to a previous 8-month follow-up study on Vietnamese adolescents [32]. This disparity can be attributed to variations in the study population and research methods. Nevertheless, the decrease in the prevalence of self-harm thoughts over time aligns with findings reported in other studies [32, 33]. One possible explanation is that individuals in the study population exhibited psychological resilience over time, indicating an intrinsic capacity to handle stressors and difficulties. The decline in thoughts of self-harm might also be associated with innate developmental shifts. As individuals navigate various life stages, their coping strategies, social support networks, and overall mental well-being could undergo changes, potentially leading to a decrease in self-harm ideation [33].

The finding in this study that having ever been pregnant was associated with having an increased chronicity of self-harm ideations echoes findings from previous studies [2, 34, 35]. This is likely because experiencing pregnancy in conflict and displacement contexts may heighten psychological distress, driven by trauma-related factors such as healthcare challenges and the difficulties of parenting in precarious environments, potentially intensifying self-harm ideations [31]. Additionally, teenage motherhood poses unique challenges for female refugees, with the added responsibilities contributing to heightened stress and emotional distress, potentially influencing the increased chronicity of self-harm ideations [36]. Limited access to reproductive healthcare, social isolation, and stigma surrounding pregnancies in challenging circumstances further compound the complex interplay of factors that may contribute to the enduring nature of self-harm ideations among teenage female refugees [31, 37].

The finding in this study that having a spouse as the head of the family reduced the chronicity of self-harm ideations suggests the potential protective role of family structure in the mental well-being of this vulnerable population. Having a spouse as the family head among female teenage refugees may offer crucial social support and emotional stability [28]. This stems from the cultivation of robust family bonds and supportive marital relationships, establishing a feeling of security [29]. The stability in family structure enhances cohesion, eases individual stressors through shared responsibilities and joint decision-making, and has a positive effect on psychosocial well-being, ultimately reducing the persistence of self-harm ideations within this vulnerable population [30].

Although our study did not find an association between adolescent age, media exposure, and education levels with self-harm, a wealth of information is available on this topic. Media exposure, especially to social media, whether intentional or accidental, is likely to elicit emotional disturbance that can lead to self-harm. Similarly, a cross-sectional study in India shows that up to 50% of adolescents exposed to social media are likely to engage in self-harm [38]. In a different context, a systematic literature review of 9 studies showed a clear association between social media use and self-harming behavior in adolescents [39]. Age is an important predictor of self-injurious behaviors in adolescents. A cross-sectional study showed that the rates of self-injury decline from age 13 to 20 years. However, this study included both male and female adolescents. A similar study among female adolescents showed a drop in self-harm as they age beyond 15 years [40]. However, these findings were not in the context of refugee settings.

### Implication and recommendations of study findings

The findings of this study highlight the imperative for specialized mental health interventions targeted at female teenage refugees, addressing the diverse levels of self-harm ideations. Tailored programs should acknowledge the distinct needs associated with acute, subacute, and chronic ideations, considering various risk factors. Recommendations include providing reproductive health support to address mental health challenges associated with pregnancy and initiating family support programs to strengthen relationships and reduce the likelihood of chronic self-harm ideations.

### Limitations

Our study’s cross-sectional design restricts the determination of causality relationships. Future research endeavors could adopt longitudinal approaches, incorporating historical data, to track changes over time and provide a more comprehensive assessment of causal associations, thereby enhancing the depth of our findings.

## Conclusions

There is varying chronicity of manifestation of self-harming ideations among female teenagers living in Refugee Settlements of Northern Uganda. Having ever been pregnant significantly increases the likelihood of chronic self-harming ideations, while having a spouse as the family head significantly decreases the likelihood. These findings underscore the importance of considering diverse demographic and familial factors in addressing the mental health needs of female teenage refugees, emphasizing the need for tailored interventions and support systems aimed at mitigating self-harm behaviors in this vulnerable population.

### Electronic supplementary material

Below is the link to the electronic supplementary material.

Supplementary Material 1

## Data Availability

The dataset is available within this manuscript (Supplementary file 1).
